# PARTIE: a partition engine to separate metagenomic and amplicon projects in the Sequence Read Archive

**DOI:** 10.1093/bioinformatics/btx184

**Published:** 2017-03-30

**Authors:** Pedro J Torres, Robert A Edwards, Katelyn A McNair

**Affiliations:** 1 Department of Biology, San Diego State University, San Diego, CA, USA; 2 Computational Science Research Center, San Diego State University, San Diego, CA, USA; 3 Department of Computer Science, San Diego State University, San Diego, CA, USA

## Abstract

**Motivation:**

The Sequence Read Archive (SRA) contains raw data from many different types of sequence projects. As of 2017, the SRA contained approximately ten petabases of DNA sequence (10^16^ bp). Annotations of the data are provided by the submitter, and mining the data in the SRA is complicated by both the amount of data and the detail within those annotations. Here, we introduce PARTIE, a partition engine optimized to differentiate sequence read data into metagenomic (random) and amplicon (targeted) sequence data sets.

**Results:**

PARTIE subsamples reads from the sequencing file and calculates four different statistics: *k*-mer frequency, 16S abundance, prokaryotic- and viral-read abundance. These metrics are used to create a RandomForest decision tree to classify the sequencing data, and PARTIE provides mechanisms for both supervised and unsupervised classification. We demonstrate the accuracy of PARTIE for classifying SRA data, discuss the probable error rates in the SRA annotations and introduce a resource assessing SRA data.

**Availability and Implementation:**

PARTIE and reclassified metagenome SRA entries are available from https://github.com/linsalrob/partie

**Supplementary information:**

Supplementary data are available at *Bioinformatics* online.

## 1 Introduction

The combination of high-throughput sequencing technologies and advanced bioinformatics techniques are rapidly accelerating genomic and metagenomic analysis (Aziz *et al.*, 2008; Meyer et al., 2008) and leading to the explosive growth of sequence data (Cochrane *et al.*, 2013; Kodama *et al.*, 2012). The NIH Sequence Read Archive (SRA) was started in 2009 and is the primary archive of high throughput sequence data (National Center for Biotechnology Information, 2009). Sequence data was deposited into the SRA at more than 10 Tbp per day in 2016 (data from https://www.ncbi.nlm.nih.gov/sra/docs/sragrowth/).

Sequence data deposited in the SRA is necessarily dependent on the submitters for accurate classification of the data. The SRA curators strive to accurately capture appropriate metadata on the deposited sequences; however, annotations are not uniform or standard leading to a variety of ways to describe samples deposited to the databases. DNA sequencing has revolutionized microbial ecology (Dinsdale *et al.*, 2008), however there are two orthogonal approaches commonly used to explore the microbial universe: amplicon where a part of a single gene (usually the 16S gene) is amplified and sequenced (Human Microbiome Project Consortium, 2012), and shotgun metagenomics (random) (Handelsman, 2004) where all the DNA is extracted and sequenced (Edwards, 2006;DeLong *et al.*, 2006). The former provides a rapid, portable and cheap method to identify the organisms in a sample, while the latter provides details about those organisms and the functions that they are performing (Dinsdale *et al.*, 2013). Unfortunately, these two techniques, which provide different data sets and require different analyses, are often included under the ‘metagenomics’ umbrella in the SRA.

We created the partition engine, PARTIE to curate metagenomics data from the SRA into amplicon (targeted) and shotgun metagenomic (random) data sets. PARTIE analyzes four aspects of the sequence file: the unique *k*-mer frequency, the abundance of 16S rRNA sequences and the prokaryotic- and viral-read abundance. We demonstrate the accuracy of PARTIE for classifying SRA data, discuss the probable error rates in the SRA annotations and introduce a resource assessing SRA data.

## 2 Materials and methods

Three sequence databases were created: a 16S rRNA database (9254 genes), a phage database (2662 genomes) and a prokaryotic genome database (1650 genomes). The 16S and prokaryotic databases were downloaded from the GenBank ftp site. The phage genomes were downloaded from the PHANTOME website.

The sra-toolkit’s fastq-dump program is used to extract the first 10 000 reads from the SRA file and to output the reads in fasta format. These reads are aligned against the three previously discussed databases using the program Bowtie2, and the percentage of reads that hit to each databases is calculated (Langmead and Salzberg, 2012). The percentage of ‘unique *k*-mer’ is also calculated for each metagenome by using the program Jellyfish to find all *k*-mer (default, *k* = 15) in the metagenome read subset, and counting those *k*-mer that appear 10 or less times (Marçais and Kingsford, 2011). This criterion relies on the observation that samples containing amplicon sequences have a high number of similar *k*-mer resulting in a decrease in unique *k*-mer abundance. Conversely, samples containing shotgun metagenomic sequences have more random sequences, and thus a wider distribution of unique *k*-mer.

The four frequency traits (16S, phage, prokaryotic, unique *k*-mer) are calculated for each of the downloaded SRA metagenomes, along with the response type (Amplicon, Other, WGS). Initially, an unsupervised RandomForest using the R library (Breiman, 2001) was used to classify the data, and then we pruned some to generate a refined classification engine.

## 3 Discussion

**Fig. 1:**
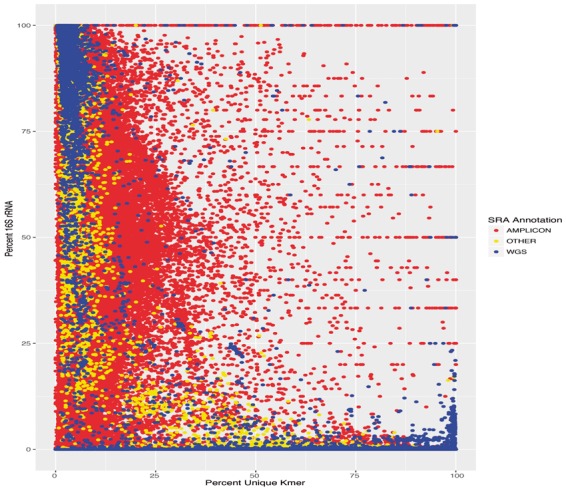
Scatter plot of percent 16S rRNA vs percent unique *k*-mer. The sequence source annotation was obtained directly from the sequence read archive (SRA) database. Eighteen different sequence source annotations were lumped into the ‘Other’ category

PARTIE was first used to calculate the parameters for 211 787 SRA datasets in which the sequencing strategy was annotated by the submitter as either Amplicon (160 247 samples), WGS (44 651 samples) or a combined data set that were classified as ‘Other’ (6889 samples). The ‘Other’ is a combination of different sequencing library construction approaches where there are too few of any individual data sets to build a robust classifier for them (Supplementary Table S1). The partition engine workflow begins by identifying all the potential metagenomes from the Sequence Read Archive. The SRA SQLite dumps from SRAdb (Zhu *et al.*, 2013) are used to identify all potential metagenome sequences. We currently identify samples where the library source is ‘METAGENOMIC’, the study type is ‘METAGENOMICS’, or where the sample's scientific name can be expanded from microbiome or metagenome. We focus on correctly classifying the whole genome shotgun (WGS) sequencing data sets, and so we filter those to remove any in which the annotators identify the library strategy as AMPLICON or PCR. The relative contribution of each of the approaches is shown in Supplementary Figure S1. Those metagenomes are downloaded using the sra-toolkit’s prefetch capability and the Aspera ascp-client (National Center for Biotechnology Information, 2009). The initial classification of these samples (Fig. 1) by the random forest resulted in a 5.4% out of bag error with the most important predictor variables being the percent unique *k*-mer sequences and the percent 16S rRNA (Supplementary Fig. S2). Random Forests also predicted that both the instrument type and read length are minor predictors of metagenome type. However, there is an uneven distribution of sequencing with different machines, with currently many more amplicon sequences generated by the Illumina

MiSeq and many more WGS data sets generated by the Illumina HiSeq 2000 (data not shown). This is not a variable that is dependent on the sequencing *per se*, and is likely to change over time, and therefore was excluded from the analysis. It was apparent from the data that the classification could be improved through manual curating. Since the fraction of unique *k*-mer was the most important predictor, a threshold value was calculated to reclassify each metagenome solely on the *k*-mer abundance. When the *k*-mer frequency data was plotted on a histogram, a distinct bimodal distribution was apparent (Supplementary Fig. S3). The centroids of the two peaks were identified using *k*-means clustering (Hartigan, 1975) resulting in a midpoint value at 47%, which was rounded to 50% for stringency and simplicity. Using this revised calculation, several questionable data sets were omitted from the training data sets. The amplicon test set was decreased by 3502 data sets to 156 745 data sets. The WGS data was decreased by 7032 data sets to 37 619 data sets and the other data sets were reduced by 7. This robust training set was used to build an automatic classification and partition engine that had a 2.45% error rate (Supplementary Table S2). The PARTIE analysis package is being used to routinely reclassify data sets from the SRA. Over 270 000 datasets have been reclassified as of March 1, 2017, and an up to date list is available at https://github.com/linsalrob/partie/. The number of data sets of each type that were reclassified is shown in the matrix in Supplementary Table S3. One fifth of the random sequencing datasets have been reclassified as amplicon projects. We also recommend examining the four calculated parameters as there are cases in which both WGS and amplicon sequencing is used (e.g. Run ID ERR162903), and no automatic partition approach will correctly classify this library.

## Funding

This work was supported by National Science Foundation grants MCB-1330800 and DUE-132809 to RAE.

*Conflict of Interest*: none declared.

## Supplementary Material

Supplementary Data

Supplementary Data

Supplementary Data

Supplementary Data

Supplementary Data

Supplementary Data
