# Supplementary figures and images for: PARTIE: a partition engine to separate metagenomic and amplicon projects in the Sequence Read Archive
Source: Bioinformatics. 2017 Mar 30;33(15):2389–91. doi: 10.1093/bioinformatics/btx184 (PMC5860118; doi:10.1093/bioinformatics/btx184)

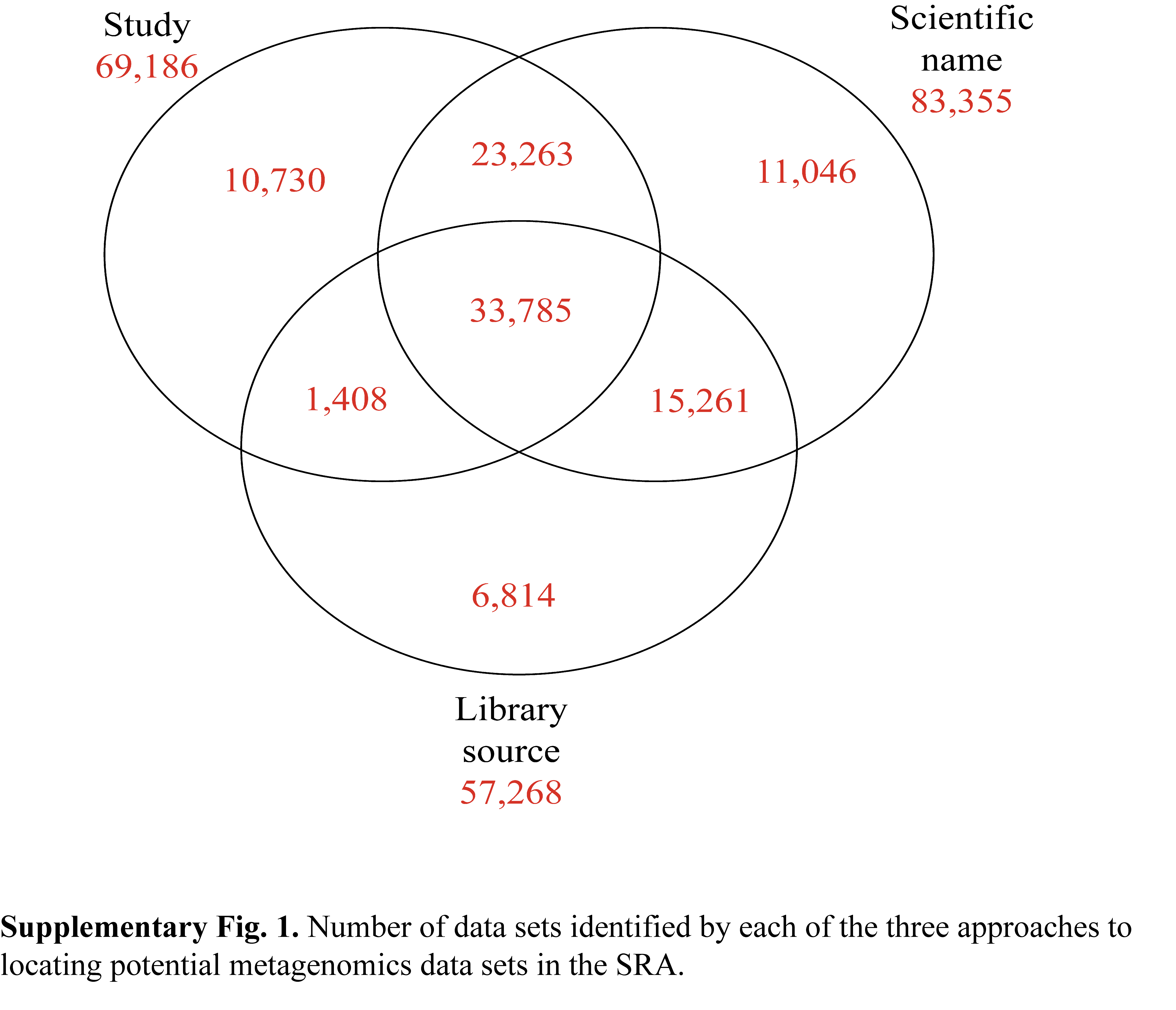

Supplement: Supplementary Data [file btx184_supplementary_fig1.png]

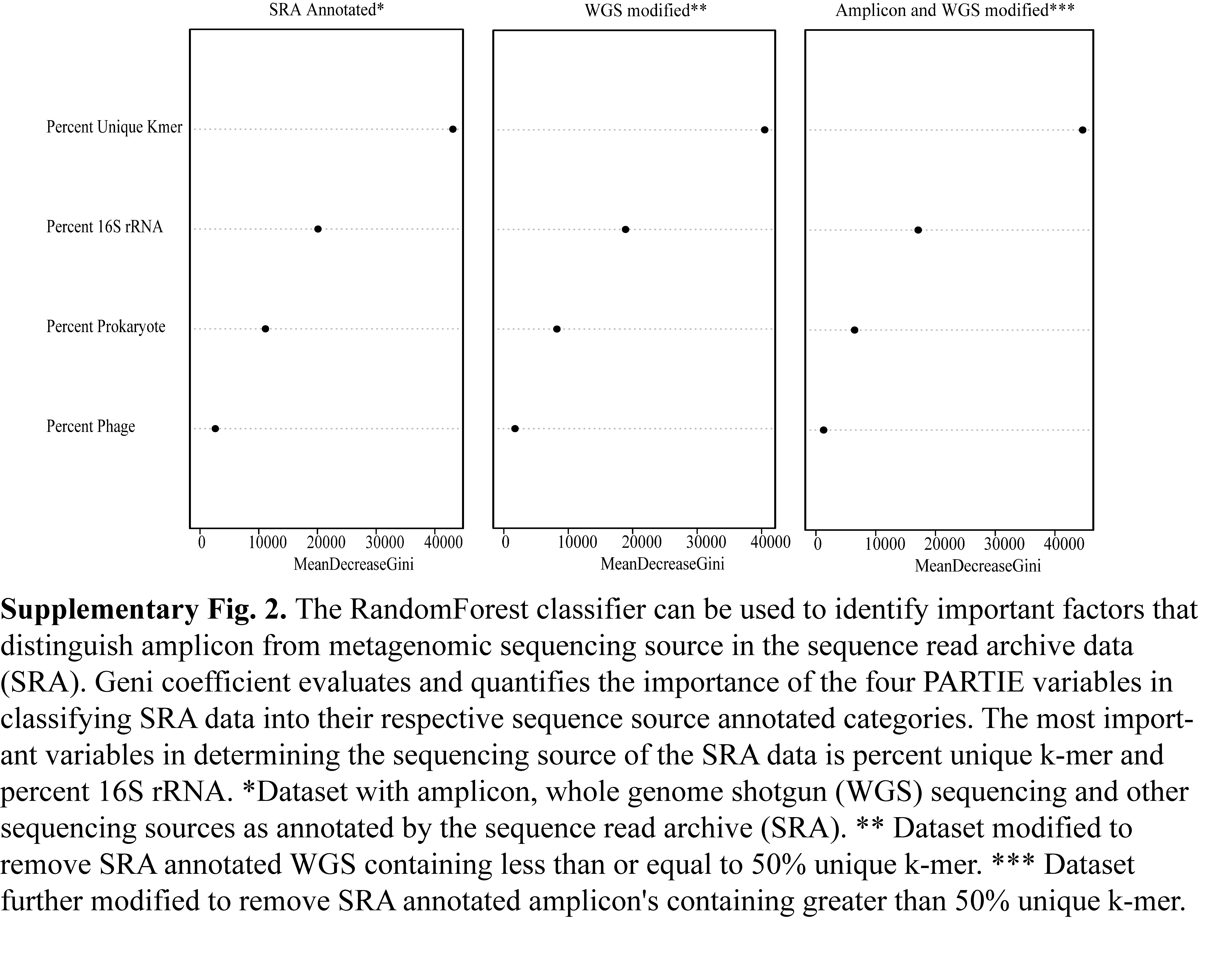

Supplement: Supplementary Data [file btx184_supplementary_fig2.png]

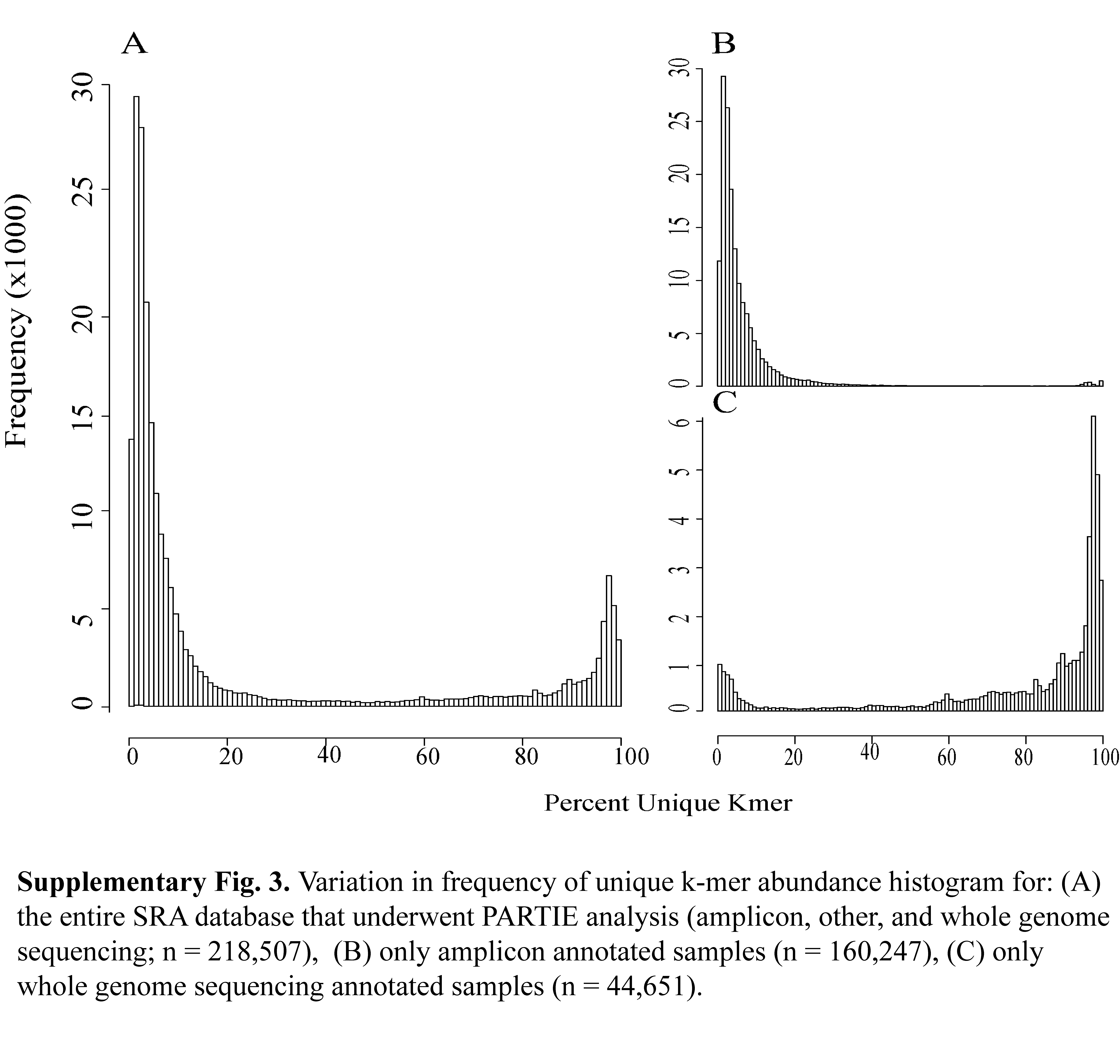

Supplement: Supplementary Data [file btx184_supplementary_fig3.png]
